# Prediction of heat stress response in dairy cows using milk mid-infrared spectra

**DOI:** 10.1038/s41598-026-39287-9

**Published:** 2026-03-19

**Authors:** Pauline Lemal, Clément Grelet, Frédéric Dehareng, Hélène Soyeurt, Martine Schroyen, Nicolas Gengler

**Affiliations:** 1 University of Liège, Gembloux Agro-Bio Tech (ULiège-GxABT), Gembloux, 5030 Belgium; 2 Walloon Agricultural Research Center (CRA-W), Gembloux, 5030 Belgium

**Keywords:** Heat stress, Dairy cows, Mid-infrared spectra, Prediction equation, Computational biology and bioinformatics, Physiology, Zoology

## Abstract

Measuring individual cows’ response to heat stress at large-scale is challenging because physiological traits are not recorded routinely, and production traits are unspecific and require environmental data for interpretation. Milk mid-infrared (MIR) spectra, already recorded in routine, offer a potential alternative, as heat stress affects milk composition and is therefore expected to be reflected in MIR spectra. This study thus aimed to develop a MIR prediction equation for individual heat stress response. Surface temperature and milk traits from 399 cows were recorded to develop a combined heat stress response phenotype. This phenotype resulted from two equations: one predicting surface body temperature (R^2^ = 0.67; RMSE = 0.64 °C) and one classifying records into three heat stress response classes based on surface temperature and milk composition (accuracy = 61%). The final prediction was applied to historical milk recording data associated with weather information to assess external validity. A mixed model was also fitted to identify cow characteristics associated with stronger predicted heat stress responses. As reported in the literature, multiparous cows, in early lactation, with the highest 24 h milk yield tended to be more affected. Overall, the prediction developed in this study shows strong potential for routine heat stress detection.

## Introduction

Heat stress is known to negatively impact cattle welfare and production. Dairy cows are especially affected due to the high heat load associated with milk production^1^. In the next decades, as a result of greenhouse gas emissions, heat stress is expected to increasingly impact global cattle populations with the earliest and most severe consequences projected in poor tropical regions^2^. This highlights the importance to find solutions to mitigate adverse effects of heat stress. In the literature, two complementary approaches are proposed: management strategies (including housing adaptation, ration modification, adapted activity scheduling, etc.) and genetic selection^3^. Efficient management practices as well as reliable genetic evaluations depend on the ability to detect animals affected by heat stress.

One of the most direct ways to assess if a dairy cow is affected by heat stress is to measure its body temperature^4,5^. However, direct detection of heat stress through physiological parameters like body temperature is difficult to implement routinely at a large-scale. Conversely, heat stress detection through production loss is adapted to routine but less specific and requires knowledge of environmental parameters^6,7^. Similarly, genetic evaluation for thermotolerance is generally based on reaction norm models fully dependent on temperature and humidity index (THI) or other environmental variables^8^. Despite this, the THI, based on temperature and humidity data generally recorded from weather stations, does not take into account potential heat stress mitigation systems such as fans, shade access, water availability, etc., nor the cows’ physiological adaptation to hot environments^7^.

A potential alternative is to use milk composition as a proxy, especially milk mid-infrared (MIR) spectra obtained through Fourier-transform mid-infrared (FT-MIR) spectroscopy during routine milk recording, to predict the individual heat stress responses of cows. Indeed, milk FT-MIR spectra are already largely utilized to predict fine milk components but also indirect phenotypes like blood metabolites, efficiency, methane emissions, etc^9^. Because milk composition is known to be affected by heat stress^10,11^, there is a great potential that milk FT-MIR spectra can also be used to predict individual heat stress responses. This approach would allow to monitor specific responses of cows to heat stress, independently of the THI, while being compatible with large-scale routine data collection. To generate reference data for the individual response to heat stress, both surface body temperature and milk traits were used. Indeed, it has been shown that surface temperature of the udder is higher in cows affected by heat stress^12,13^. Although only partial correlations have been reported between milk yield and body temperature^14^, it indicates that surface temperature and milk traits capture different and complementary aspects of the response to heat stress.

On this basis, the objective of this study was to develop a first proposition of milk FT-MIR-based prediction equation for individual heat stress response in dairy cows.

## Materials and methods

This study did not involve any experimental procedures on live animals. Milk recording data were collected by the Walloon Breeders Association (awé groupe – Elevéo, Ciney, Belgium) as part of routine herd management in farms enrolled in official milk recording programs in the Walloon region of Belgium and therefore fall within the scope of non-experimental agricultural practices. Body temperature measurements were obtained using infrared thermography by the University of Liège – Gembloux Agro-Bio Tech (Gembloux, Belgium) in five commercial dairy farms, during routine milking sessions and without additional handling or restraint beyond standard milking procedures. Although this activity is not part of standard agricultural practice, it was performed without any procedures likely to cause pain, suffering, distress, or lasting harm equivalent to, or higher than, that caused by the introduction of a needle in accordance with good veterinary practice. Therefore, this study does not meet the criteria defined by Directive 2010/63/EU for procedures considered as animal experimentation, and did not require ethical approval.

### Weather data

Hourly temperature (T) and relative humidity (RH) were extracted from the Agromet platform^15^ that collects weather information from the Pameseb network which consists in 30 weather stations distributed across Wallonia and separated by approximately 30 km. All records from every farm of this study were associated with weather data from the closest weather stations to the farms. Additionally, 5 farms were equipped with Tinytag TGP-4500 dataloggers (Gemini Data Loggers Ltd., UK) to collect in-barn hourly T and RH. Weather station and in-barn hourly THI were then calculated using the following formula^16,17^:

$$\:THI=\left(1.8\times\:T+32\right)-[\left(0.55-0.0055\times\:RH\right)\times\:\left(1.8\times\:T-26\right)]$$

Hourly THI where combined in daily THI by computing the mean of the 24 THI values of a given day. To take into account the delay between the onset of heat stress and the effects on cows, the average THI from the test-day and the 3 previous days ($TH{I_{\overline {{td - 3d}} }}$) were calculated and rounded to the closest integer^18^.

### Surface temperature data

**Fig. 1:**
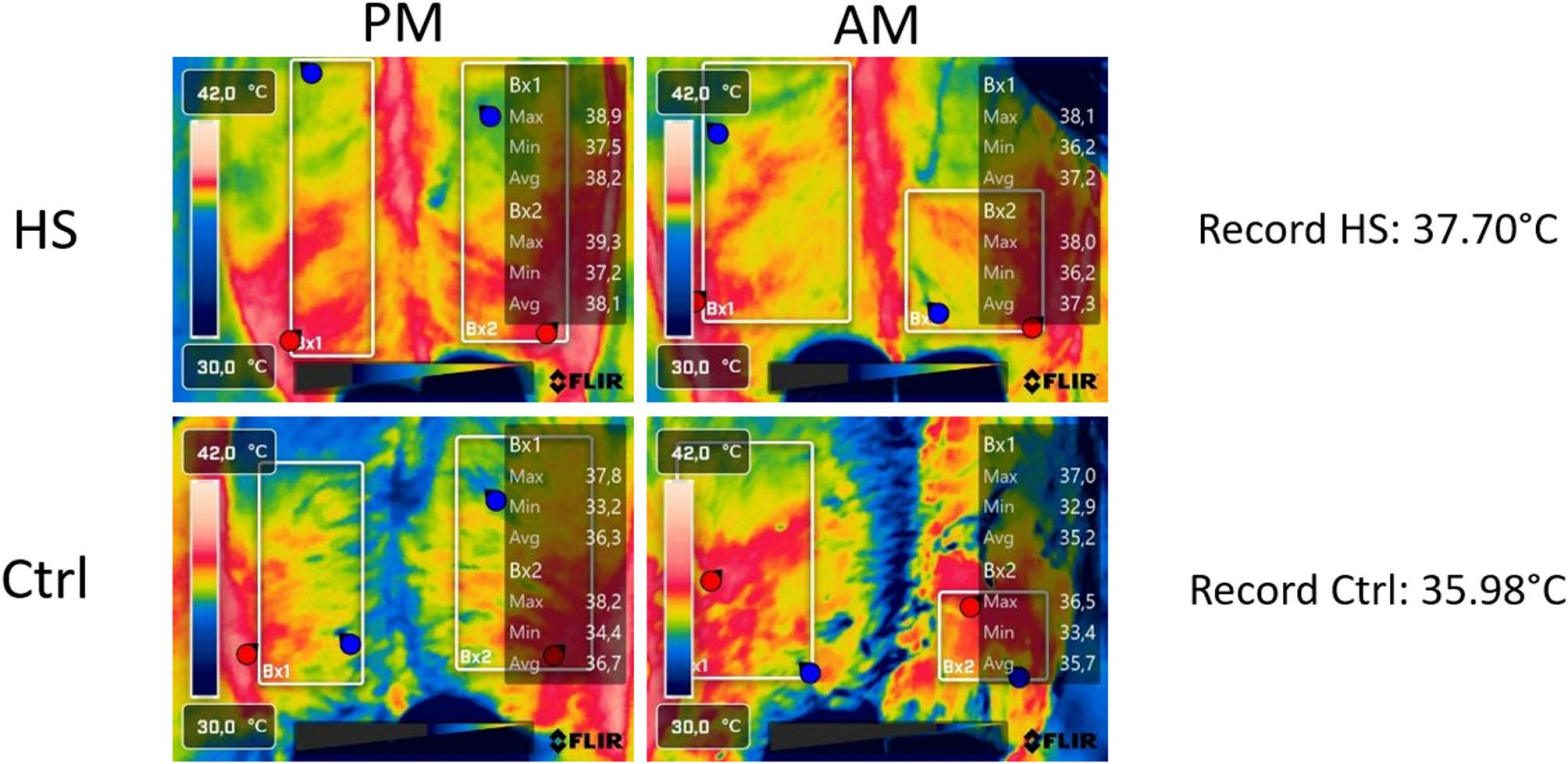
Surface body temperatures measured with an infrared FLIR E5 Pro camera on the udder of the same cow during PM and AM milkings (once during a heat wave (HS) and once during a thermoneutral period (Ctrl)). The temperature of the two quarters were averaged to obtain one temperature per image and the temperature of the two images of the same milk recording (PM and AM) were averaged to obtain one record per milk recording (on the example: 37.70 °C for the recording during the heat wave and 35.98 °C for the recording during the thermoneutral period).

In the 5 farms equipped with Tinytag TGP-4500 dataloggers (Gemini Data Loggers Ltd., UK) in barns, a FLIR E5 Pro camera (Teledyne FLIR, USA) was used to obtain infrared images of individual cow’s udders during milking simultaneously with routine milk recording data collection. Surface temperature of the udder was chosen instead of other body temperature recording methods because it is both faster and non-invasive, allowing recordings in commercial farms. Indeed, although it requires an additional operator during milk recording and a subsequent time-consuming image processing, this method does not interfere with the normal activities of the farm. Two images of the rear udder were taken per animal and per test day, one during the evening milking and the second during the next morning milking. The average surface temperatures of the two visible quarters were obtained by manually selecting the regions of interest on each image with the FLIR Thermal Studio software (version 2.0.53, Teledyne FLIR, USA), ensuring only clean skin areas were included. The central area between the quarters and the edges of the udder in contact with the hind legs were excluded to prevent an overestimation of the surface temperature due to friction (Fig. 1). Ambient temperature and relative humidity were entered into the software during image analysis to account for environmental variations between the different measurement periods. Emissivity was set on ε = 0.95 for all images. Surface temperature values for the two images of a given cow for a given test day were averaged to have one record per animal and per test day. A total of 679 records were obtained with this method on 399 cows from 5 farms. Each farm was visited two times the same year: (1) during a summer heat waves (a distinct heat wave for each farm) (total of 350 records) (June, July or August) and (2) during a thermoneutral period (total of 329 records) (October).

To ensure that udder surface temperature was not increased independently of heat stress by subclinical mastitis, temperatures recorded during the thermoneutral period were compared between cows with somatic cell count (SCC) ≥ 200,000 cells/mL and SCC < 200,000 cells/mL. No significant difference was observed (≥ 200,000 (*n* = 54): 34.96 ± 0.77 °C; < 200,000 (*n* = 275): 35.09 ± 0.64 °C; t-test, *p* = 0.19) suggesting that surface temperature records from cows with high SCC can be used in the analysis without bias.

Only data from Holstein cows were considered for this study. The lactation numbers of the recorded cows ranged from 1 to 9 and the days in milk (DIM) from 5 to 610. The repartition of cows in the five farms is reported in Table 1.

**Table 1:** Distribution of sampled animals for surface body temperature simultaneously with a milk recording. Data collected during a heat wave are shown in the HS columns and data recorded during a thermoneutral period are presented in the Ctrl columns.

Lactation number	DIM	Farm									
		1		2		3		4		5	
		HS	Ctrl	HS	Ctrl	HS	Ctrl	HS	Ctrl	HS	Ctrl
1	5–30	1	0	2	0	0	0	0	0	0	0
	31–100	4	0	2	2	0	0	3	1	0	0
	101–200	6	6	7	5	4	0	5	2	2	0
	201–365	6	8	10	8	11	8	20	10	12	9
	> 365	1	0	0	2	1	2	2	3	1	0
2	5–30	2	0	4	0	2	2	0	2	0	1
	31–100	4	5	5	8	5	9	3	8	0	1
	101–200	0	6	10	6	11	6	6	6	5	0
	201–365	5	1	7	9	8	12	13	9	8	8
	> 365	0	0	1	0	1	2	1	2	1	3
3+	5–30	4	2	3	1	6	2	1	1	0	0
	31–100	2	8	6	8	7	12	14	10	0	2
	101–200	9	5	5	5	4	12	17	15	3	0
	201–365	9	12	12	10	13	7	17	26	7	6
	> 365	1	0	0	0	2	4	4	6	2	3
Total		54	53	74	64	75	78	106	101	41	33

### Milk recording data and residual analysis

All milk samples were collected in the frame of milk recording according to the guidelines edited by the International Committee for Animal Recording (ICAR Dairy Cattle Milk Recording Working Group, 2017) and with ICAR-approved milk samplers. Samples were analysed at the Comité du Lait (Battice, Belgium) on FT + and FT7 Foss instruments (Foss, Hillerød, Denmark). All the FT-MIR spectra from the different instruments were standardized to be merged into a common dataset following the procedure described in Grelet et al. (2015)^19^. Milk yield, fat percentage, protein percentage, and standardized FT-MIR spectra were extracted from routine milk recording database. Magnesium (Mg) concentrations were obtained by applying the prediction equation described in Christophe et al. (2021)^20^ on standardized FT-MIR spectra. This trait was added to the classical production traits because it was identified as a potentially good biomarker of heat stress in a previous study^18^.

Residuals were estimated from historical milk recording data from January 2019 to October 2024 of the 5 farms recorded for surface body temperature which represents a total of 24,384 test-day records. These historical data were used to fit the following single-trait random regression model and obtain residuals for the heat‑wave days (Eq. 1):

1 $$\:\mathbf{y}=\mathbf{X}\mathbf{b}+\mathbf{Q}\left({\mathbf{Z}}_{1}\mathbf{a}+{\mathbf{Z}}_{2}\mathbf{p}\right)+\mathbf{e}$$

with **y** the vector of test-day values for the trait of interest (milk yield, fat percentage, protein percentage or Mg concentration); **b** the vector of fixed effects including the herd × year of recording, the month of recording, the class of lactation number (3 classes: 1, 2, 3+) and the class of days in milk (DIM) (19 classes: 5–10, 11–15, 16–20, 21–25, 26–30, 31–40, 41–50, 51–60, 61–70, 71–80, 81–90, 91–100, 101–120, 121–150, 151–200, 201–250, 251–305, 305–365, > 365 DIM); **a** the vector of coefficients for the additive genetic random effect; **p** the vector of coefficients for the permanent environment random effect; **X**, **Z**_**1**_ and **Z**_**2**_ the matrices linking observations and effects, **Q** the covariate matrix containing second degree Legendre polynomials for standardized DIM ($\:P0=1$, $\:P1=x$, $\:P2=\frac{1}{2}\times\:{(3x}^{2}-1)$ with $\:x=\frac{2\:\times\:(DIM-5)}{365-5}-1$ with a maximal value set at 1 for DIM higher than 365) and **e** the vector of residuals.

Residuals for milk yield, fat percentage, protein percentage and Mg concentration were estimated in addition to the surface temperature measurements described previously to determine if the cows were in heat stress during the summer heat wave recordings. Indeed, by fitting a model correcting for systematic effects such as the lactation stage or the recording period without including a THI effect, the effect of heat stress can be observed in the residuals without the impact of confounding effects^18^. When residual values are lower than 0, the recorded phenotypes can be considered as lower than expected for a cow in normal conditions with the corresponding characteristics and environment.

To enable comparison between traits, residuals were expressed as standardized residuals by dividing them by their standard deviation (SD).

### Definition of heat stress response phenotypes

The first heat stress response phenotype of this study was exclusively based on the surface body temperature values recorded with an infrared camera while, for the second heat stress response phenotype, cows were classified as affected by heat stress when both an increase of surface body temperature and a modification of milk composition were detected. A surface body temperature record was considered abnormal when it was higher than the mean + 3SD of the temperatures recorded during the thermoneutral day of the corresponding farm. Based on the results of this study and a previous study^18^, milk composition was considered affected by heat stress when standardized residuals for protein percentage and Mg concentration were both lower than − 0.1. The limit was not set to 0 to take into account the potential normal slight fluctuation of these traits. On the other hand, cows presenting during the summer heat wave recording a temperature lower than mean + 2SD compared to the thermoneutral day and residuals for both protein percentage and Mg concentration higher or equal than − 0.1 were classified as non-affected by heat stress.

### Model calibration

A pre-treatment was performed on the standardized milk FT-MIR spectra by applying a first derivative with a gap of 5 and by selecting the spectral regions having a low noise signal. The derivative at each wavenumber (λ) was calculated by subtracting the absorbance at λ + 2 from the absorbance at λ-2. From the 1,060 wavenumbers in the FT-MIR spectra, 1056 derivative values were obtained (from wavenumber 3 to 1058). From those first-derivative values, 212 wavenumbers were kept for the next steps of the analysis in the regions 968.09–1,577.49 cm^−1^, 1,731.76–1,762.62 cm^−1^, 1,781.90–1,808.90 cm^−1^, 2,830.99–2,965.98 cm^−1^ (point numbers 12–170, 210–218, 223–230, 495–530 based on the 1060 original points).

To predict the surface body temperature (first heat stress response phenotype), a partial least square (PLS) regression was performed on the 679 records measured both during heat waves and thermoneutral periods. Only spectral data was used as predictor variables. To reduce noise before to run the PLS, a pre-selection of the most correlated variables to the phenotype of interest was performed. In this case, only wavenumbers with a correlation of at least 0.3 with surface body temperature were kept for a total of 102 wavenumbers. A 5 folds cross-validation was done by randomly dividing the reference dataset in 5 groups. As cows were only recorded once during the heat stress periods and once during the thermoneutral periods, each cow-temperature period could not be both in calibration and in validation datasets during the cross-validation. Instead of excluding individual cows as commonly practiced, the cow-temperature period was used. This approach was chosen due to the contrast observed across environmental conditions. Indeed, a given cow presented opposite surface body temperature values depending on the thermal period. Thus, incorporating the same cow in both calibration and validation, but associated with distinct periods, may help to identify condition-dependent signals rather than individual-specific characteristics. We tested between 1 and 50 latent variables and retained 42 components based on the best predictive performance (highest cross-validation coefficient of determination (R^2^_cv_) and lowest cross-validation root mean square error (RMSE_cv_)).

To classify milk samples as affected by heat stress or non-affected by heat stress (second heat stress response phenotype), a random forest (RF) analysis was performed. Spectral data were used as predictor variables as well as the month of recording formatted in a dummy variable according to the risk of heat stress (October-Mars: month without risk of heat stress, April-September: month with risk of heat stress). As for the PLS analysis, a pre-selection of variables was performed. A first RF was used to identify the 50 most important variables which were retained for the final RF analysis. The RF was implemented using the ‘ranger’ package in R (version 4.4.2), applying default parameters (500 trees, 26 predictor variables randomly selected at each split (i.e., mtry), minimal terminal node size of 1, and the Gini index as splitting criterion). From records measured during heat waves, a total of 88 records were classified as affected by heat stress (X1) and 28 as non-affected by heat stress (X0) based on the criteria described previously. To have an equal distribution of records among groups and to provide data from other months of the year, 60 records, randomly selected from milk recording performed in the same farms outside the summer months (exclusion of June, July and August recordings) and when the $TH{I_{\overline {{td - 3d}} }}$ was lower than 60, were added to the non-affected by heat stress group (X0). The limit was set to 60 because it is the minimal heat stress THI threshold well represented in literature^21–23^. A third intermediate group (X0.5) was created with 88 records obtained during the heat wave recordings from cows classified as neither in the affected group (X1) nor in the non-affected group (X0). Those records were randomly selected among the 234 cows that did not meet the criteria to be classified as affected or non-affected by heat stress. This intermediate category was included to reflect the grey zone between the affected and non-affected groups, representing animals with uncertain heat stress status. Including this class helped improve the separation between the two extremes. Performance of the discriminant model were expressed in terms of sensitivity (percentage of good classification in the high content group), specificity (percentage of good classification in the low content group) and global accuracy (global percentage of correct classification).

### Combination of models and large-scale application

A total of 1,038,149 milk standardized FT-MIR spectra from routine milk recording of Walloon Holstein cows from January 2020 to December 2022 were extracted to apply the developed models on external data. All records were associated with weather data from the closest weather stations to the farms and $TH{I_{\overline {{td - 3d}} }}$ were calculated as described previously.

Both prediction models were applied on the spectra. To increase the chance to really target heat stress and reduce the inherent errors of both models, a combination of the two models was also proposed. Records with a predicted surface body temperature of at least 36 °C and classified in the affected by heat stress group (X1) were considered as affected by heat stress with a value of 1, records with a predicted surface body temperature of at least 36 °C and classified in the intermediate group (X0.5) were considered as affected by heat stress with a value of 0.5 and the other records were considered as non-affected by heat stress (value of 0). On this basis, the resulting combined heat stress response phenotype presented three possible values (0, 0.5 and 1).

To evaluate the relevance and functionality of the models, the average values by $TH{I_{\overline {{td - 3d}} }}$ units were represented (Fig. 3).

In addition, to determine the characteristics of the cows with the highest risk of negative response to heat stress based on fixed effect solutions, the following mixed model was fitted on the combined heat stress response phenotype after transformation into Snell scores (Eq. 2):

2 $$\:{y}_{jklmn}={HTD}_{j}+{lact}_{k}+{DIM}_{l}+{milk}_{m}+{a}_{n}+{pe}_{n}+{e}_{jklmn}$$

with y_jklmn_ the trait of interest (combined heat stress response phenotype transformed into Snell scores); HTD_j_ the categorical fixed for herd test-day (HTD) j; lact_k_ the categorical fixed effect for the class of lactation number k (3 classes: 1, 2, 3+); the categorical fixed effect for the class of days in milk (DIM) l (classes of 5 DIM with a maximum of 365 DIM); the categorical fixed effect for the class of 24 h milk yield m (7 classes: <15 kg, ≥ 15 kg and < 20 kg, ≥ 20 kg and < 25 kg, ≥ 25 kg and < 30 kg, ≥ 30 kg and < 35 kg, ≥ 35 kg and < 40 kg, ≥ 40 kg); a_n_ the additive genetic random effect for animal n; pe_n_ the permanent environment random effect for animal n and e_jklmn_ the residual.

To fit this model, only records from HTD with at least 10 records, with associated DIM between 5 and 365 and with 24 h milk yield of at least 3 kg were kept for a total of 942,855 records. No selection was performed to only include records obtained during potential heat stress conditions in order to avoid setting an arbitrary threshold. The categorical combined heat stress response phenotype was transformed into Snell scores^24^ to achieve normalization as much as possible. Indeed, the frequency distribution across the three classes was very inequal with 94% classified as 0, 4% classified as 0.5 and 2% classified as 1. On this basis, the Snell score was 0.52 for records classified as non-affected by heat stress (value of 0), 1.80 for records classified as affected by heat stress with a value of 0.5 and 3.06 for records classified as affected by heat stress with a value of 1. The detailed method to obtain Snell score values is reported in Markey et al. (2025)^25^.

## Results and discussion

### Evidence of heat stress

The first step to develop a FT-MIR prediction equation to detect heat stress responses is to ensure that the milk samples were taken on heat stress days. For this purpose, the THI from the closest weather stations and from the barns were used as well as animal indicators including the average surface body temperature and the average standardized residuals for production traits and Mg concentration. THI values are reported in Table 2 and average individual values can be found in Table 3.

**Table 2:** Daily THI of the test-day (THI_td_), the day before the test-day (THI_d−1_), two days before the test-day (THI_d−2_), three days before the test-day (THI_d−3_) and the average daily THI of the test-day and the 3 previous days rounded to the closest integer ($TH{I_{\overline {{td - 3d}} }}$) for the 5 farms during the heat wave recordings. THI values from the closest weather stations and from inside the barns are reported.

		Farm 1	Farm 2	Farm 3	Farm 4	Farm 5
Stations	THI_td_	69.03	74.00	63.89	68.45	69.95
	THI_d−1_	65.94	66.82	63.32	66.35	70.90
	THI_d−2_	62.66	64.33	62.06	63.87	65.21
	THI_d−3_	61.54	67.38	63.93	61.13	61.16
	$TH{I_{\overline {{td - 3d}} }}$	65	68	63	65	67
Barns	THI_td_	73.14	75.48	68.70	75.27	73.47
	THI_d−1_	71.44	70.56	68.32	72.52	73.83
	THI_d−2_	68.54	69.00	67.06	71.03	69.77
	THI_d−3_	66.92	69.40	68.44	69.49	66.86
	$TH{I_{\overline {{td - 3d}} }}$	70	71	68	72	71

**Table 3:** Mean (± standard deviation) of the surface body temperature of the udder for the 5 farms visited during the heat wave recording (HS) and during the thermoneutral recording (CTRL). Average standardized residuals for milk yield, fat percentage (fat %), protein percentage (protein %) and magnesium concentration (Mg [ ]) during the heat wave recording.

	Farm 1	Farm 2	Farm 3	Farm 4	Farm 5	All
T HS (°C)	36.35 (± 0.73)	37.49 (± 0.51)	36.11 (± 0.64)	36.86 (± 0.53)	36.90 (± 0.58)	36.76 (± 0.76)
T Ctrl (°C)	35.05 (± 0.57)	35.05 (± 0.47)	34.65 (± 0.62)	35.19 (± 0.67)	35.78 (± 0.50)	35.07 (± 0.67)
r milk yield	0.07	0.11	-0.04	-0.30	-0.11	-0.08
r fat %	0.28	0.01	0.29	-0.17	-0.14	0.04
r protein %	0.12	-0.27	-0.50	-0.82	-0.28	-0.43
r Mg [ ]	-0.11	-0.50	-0.54	-0.97	-0.63	-0.61

THI from weather stations were always lower than barn THI suggesting that using weather stations THI could underestimate the impact of heat stress on cows, at least, when no specific heat stress mitigation equipment is used. In Belgium and surrounding regions, a THI threshold of around 62 is generally considered for the onset of heat stress^26^. During the heat wave recordings, weather station THI were above that threshold and in-barn THI were largely above that threshold.

Concerning surface body temperature, Garner et al. (2017)^12^ obtained an average udder surface temperature of 39.8 °C during a heat challenge in controlled-climate chambers and an average udder surface temperature of 35.2 °C in thermoneutral conditions. The average surface temperature in thermoneutral conditions was similar in this study (35.07 °C) despite the use of different devices to measure udder surface temperature. The temperature obtained in the current study during heat waves was lower (36.76 °C) which is expected because this value is calculated based on the average of evening and morning temperatures (THI generally decreased over the night during the experiment) while the THI was always higher than 74 in the controlled chambers. To take into account differences in housing and thermometer devices across studies, the temperature recorded during heat waves in this study were compared to the thermoneutral temperature records of the same farms. Indeed, even across the five farms, the time of milking varied from one farm to another. For all farms, the average surface body temperatures were highly significantly different (t-test, *p* < 0.001) between heat wave and thermoneutral recordings.

For milk traits, the residual analysis allowed to determine if the values recorded during heat waves were higher or lower than expected compared to thousands of historical milk recordings records^18^. Globally, a slightly negative average standardized residual value was obtained for milk yield. By examining each farm individually, the values were very different with negative, neutral or slightly positive average residual values. This important variability across farms was even more present for fat percentage standardized residuals leading to a global value close to 0. Conversely, standardized residuals for protein percentage were negative for all farm except one and standardized residuals for Mg concentration were negative for all farms. This is consistent with the results of a previous study^18^ that highlights Mg concentration and protein percentage as the most promising biomarkers of heat stress compared to several milk traits including fat percentage and milk yield.

All farms visited during heat waves presented THI values above 62, an increase in average surface body temperature of the cows, and negative average standardized residual values for individual Mg concentration suggesting heat stress conditions. In addition, all farms except farm 1 presented negative average standardized residual values for protein percentage.

### Individual responses to heat stress

As reflected by herd averages, farm 1 presented the lowest proportion of individuals classified as affected by heat stress (0.07) and the highest proportion as non-affected by heat stress (0.22) based on the criteria described previously. The results for all the farms individually and the global proportions are reported in Table 4. The farm with the highest proportion of cows classified as affected by heat stress during the heat wave visit was farm 2, with a proportion of 0.51 in the affected by heat stress group and 0.00 in the non-affected by heat stress group. Cows of this farm also almost all (0.96) presented an increase in body temperature higher than 3SD of the thermoneutral recording. However, although it presented a high proportion of animals with residuals lower than − 0.1 for protein percentage and Mg concentration, it did not have the highest values, as those were observed in farm 4. None of the visited farms were equipped with heat stress mitigation devices such as fans or sprinklers. The differences observed between farms even when THI were similar may be linked to varying shade access on pasture, genetic backgrounds or general management practices. It may also be due to the fact that each farm was recorded during distinct heat wave events of varying intensity across the summer, resulting in different cumulative heat load and different opportunities for acclimatation. In general, a lot of cows were classified in the intermediate group and a low proportion of animals presented no signs of heat stress at all and were classified as non-affected by heat stress. Indeed, among the 350 cows recorded during heat wave periods, 146 presented an increase of body temperature higher than mean + 3SD of the temperatures recorded during the thermoneutral day, 202 had residuals lower than − 0.1 for protein percentage and magnesium concentration and 88 showed both effects. Conversely, 95 cows presented a temperature lower than mean + 2SD compared to the thermoneutral day, 73 had residuals higher than − 0.1 for protein percentage and magnesium concentration and 28 showed the combination of both. A total of 234 cows were thus classified in the intermediate group during the heat wave recordings.

Those results confirm the idea of a great variation of responses observable for cows affected by the same stressor. In addition, the responses across farms were sometimes very different even with similar THI values, highlighting the influence of genetic background and other environmental conditions.

**Table 4:** Proportion of cows during the heat wave recordings with a udder surface temperature higher than mean + 3SD of the thermoneutral recordings (T HS (°C) > 3SD T Ctrl (°C)), with a udder surface temperature during the heat wave recordings lower than 2SD of the thermoneutral recordings (T HS (°C) < 2SD T Ctrl (°C)), with a standardized residual value lower than − 0.1 for protein percentage (r protein % < -0.1), with a standardized residual value lower than − 0.1 for magnesium concentration (r Mg [ ] < -0.1), classified as ‘affected by heat stress’ (T HS (°C) > 3SD T Ctrl (°C) and r protein % < -0.1 and r Mg [ ] < -0.1) and classified as ‘non-affected by heat stress’ (T HS (°C) < 2SD T Ctrl (°C) and r protein % ≥ -0.1 and r Mg [ ] ≥ -0.1).

	Farm 1	Farm 2	Farm 3	Farm 4	Farm 5	All
T HS (°C) > 3SD T Ctrl (°C)	0.30	0.96	0.25	0.27	0.27	0.42
T HS (°C) < 2SD T Ctrl (°C)	0.39	0.00	0.37	0.28	0.39	0.27
r protein % < -0.1	0.33	0.58	0.65	0.85	0.63	0.65
r Mg [ ] < -0.1	0.48	0.76	0.65	0.86	0.76	0.72
‘Affected by heat stress’	0.07	0.51	0.19	0.23	0.20	0.25
‘Non-affected by heat stress’	0.22	0.00	0.15	0.02	0.07	0.08

### FT-MIR predictions of heat stress response phenotypes

**Fig. 2:**
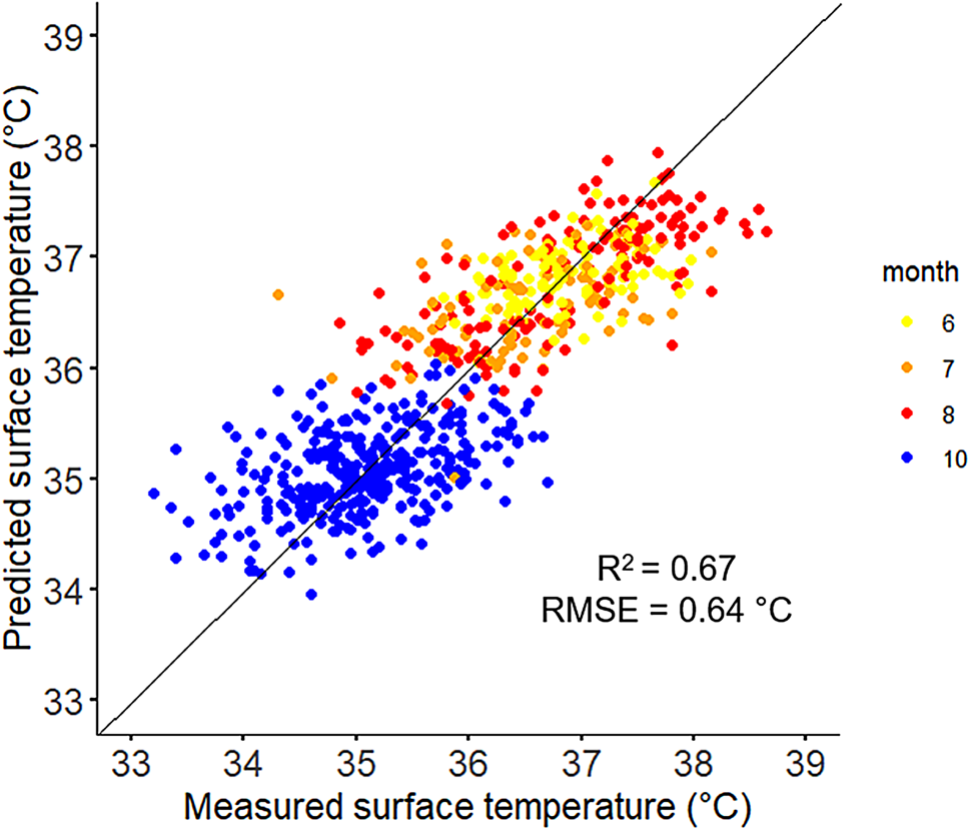
Relationship between measured surface temperature (°C) and predicted surface temperature (°C) from FT-MIR spectra (*n* = 679). The different colours represent the months of recording.

As explained previously, the first heat stress response phenotype in this study was based on the 679 surface body temperature reference records. The cross-validation coefficient of determination (R^2^_cv_) was of 0.67 and the cross-validation root mean square error (RMSE_cv_) was of 0.64 °C. The relationship between measured surface temperature and predicted surface temperature is represented in Fig. 2. These findings need to be considered as a proof of concept and need to be validated through a real validation schema with a higher number of records. However, these preliminary findings, with an error in prediction of only 0.64 °C of the body temperature (varying in this study from 33.20 to 38.65 °C) suggest a real potential of the methodology to provide information on heat stress response of individual dairy cows.

The second heat stress response phenotype was based on the classification in 3 groups: affected by heat stress group (X1), non-affected by heat stress group (X0) and intermediate group (X0.5). The global accuracy of the classification was of 61%. The confusion matrix is represented in Table 5. Cows affected by heat stress were classified with a sensitivity of 67% and a specificity of 73%. Cows non-affected by heat stress were classified with a sensitivity of 64% and a specificity of 96%. The error of predictions was principally between X1 and X0.5 which is expected because some individuals classified in the intermediate group (X0.5) were probably affected by heat stress without presenting all the signs. Conversely, cows classified as affected by heat stress (X1) were almost never misclassified by the model as non-affected by heat stress (X0).

**Table 5:**
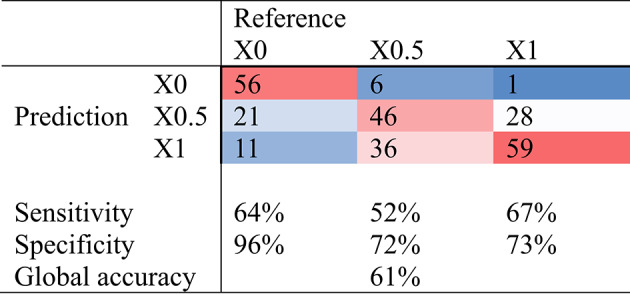
Heat map confusion matrix of predicted heat stress response group from FT-MIR spectra. X1: affected by heat stress group, X0: non-affected by heat stress group, X0.5: intermediate group.

### Combination of models and large-scale validation

**Fig. 3:**
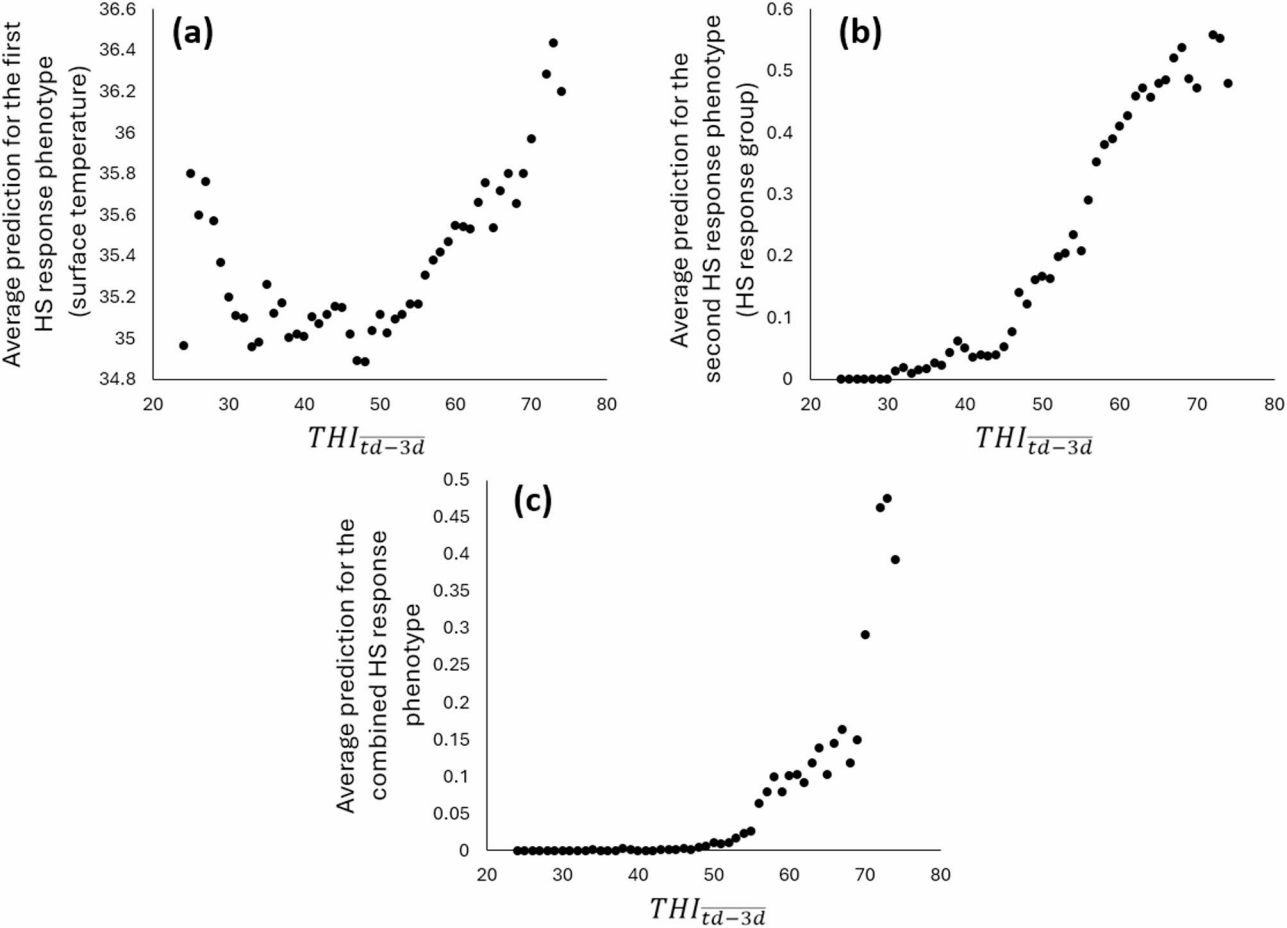
Average prediction values per class of THI (mean of the THI of the test-day and the three previous days ($TH{I_{\overline {{td - 3d}} }}$)) for the two models developed (**(a)** prediction of the udder surface temperature; **(b)** prediction of the heat stress response group) and **(c)** the combination of the two models (combined heat stress response). Only THI classes with at least 1000 records are represented. HS: heat stress.

To determine if the models developed can be used on a larger-scale, they were applied to milk recording data from other farms during another period. The predictions obtained were first represented as the averaged value per $TH{I_{\overline {{td - 3d}} }}$ in Fig. 3 (a-b). The prediction for the first heat stress response phenotype (prediction of surface temperature) showed the highest body temperature predictions when the $TH{I_{\overline {{td - 3d}} }}$ is the highest with the increase starting at a $TH{I_{\overline {{td - 3d}} }}$ of 55–60. However, an increase of the average prediction was also observable with the lowest $TH{I_{\overline {{td - 3d}} }}$ values. This could be due to similar milk composition variation in hot and cold environments compared to a thermoneutral environment. It could also be linked to management decisions. Indeed, when the weather is cold, cows are kept inside, sometimes in small and enclosed stables which could trigger responses in milk similar to those observed during heat stress. Conversely, for the prediction of the second heat stress response phenotype (prediction of heat stress response group), the average values were equal to 0 until a $TH{I_{\overline {{td - 3d}} }}$ of 30. The first records with a value of 1 or 0.5 (classified in X1 or in X0.5 by the model) started to appear after that value and reach in average 0.1 around a $TH{I_{\overline {{td - 3d}} }}$ of 50. Average values of 0.5 were obtained around a $TH{I_{\overline {{td - 3d}} }}$ of 65 and then stayed quite stable with no additional increase with the highest $TH{I_{\overline {{td - 3d}} }}$ values. On this basis, both models had complementary advantages. The first model presented a constant increase of the average prediction with increasing $TH{I_{\overline {{td - 3d}} }}$ values without the appearance of a plateau but the second model proposed values equal or close to 0 when the conditions were the coldest.

To take advantages of both models, a combination was proposed and represented in Fig. 3 (c). With this combined heat stress response phenotype, values equal or almost equal to 0 were obtained until a $TH{I_{\overline {{td - 3d}} }}$ of 50–55 and an average value of 0.1 was only reached at a $TH{I_{\overline {{td - 3d}} }}$ of 60. This suggests that the combined prediction was the best to exclude records non-affected by heat stress. For $TH{I_{\overline {{td - 3d}} }}$ values between 60 and 70, only a slight increase of the average prediction value was observed while a drastic increase was obtained from a $TH{I_{\overline {{td - 3d}} }}$ of 70. On this basis, animals started to react to heat stress at $TH{I_{\overline {{td - 3d}} }}$ around 55–60 which is close to the lowest thresholds found in literature^21–23^ which can be expected in a temperate region such as Belgium. However, the drastic increase of positive records happened around a $TH{I_{\overline {{td - 3d}} }}$ of 70 which is closer to THI thresholds proposed for hot regions^27,28^. This trend with two successive increases of the average prediction could explain the threshold differences between hot and cooler regions. Indeed, the first increase could be considered as not having a sufficient impact on production traits in average in hot countries because the second and more drastic increase is often observed. This first increase could even not exist in hot climates because animals are more adapted to hot conditions or already indirectly selected to avoid too frequent productivity losses due to heat stress.

**Fig. 4:**
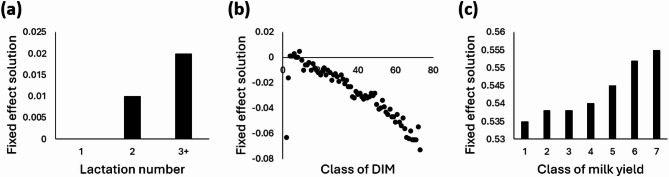
Fixed effect solutions for **(a)** lactation number (1, 2, 3+), **(b)** class of days in milk (DIM) (classes of 5 DIM with a maximum of 365 DIM) and **(c)** class of 24 h milk yield (1: <15 kg, 2: ≥15 kg and < 20 kg, 3: ≥20 kg and < 25 kg, 4: ≥25 kg and < 30 kg, 5: ≥30 kg and < 35 kg, 6: ≥35 kg and < 40 kg, 7: ≥40 kg) from Eq. 2 applied on Snell scores of the combined heat stress response phenotype.

To evaluate the functionality of the combined heat stress response phenotype, in addition to looking at the average value per THI unit, a model containing fixed effects for cow characteristics was fitted. The solutions are represented in Fig. 4. The results show that cows with higher lactation number are more susceptible to have higher values for the combined heat stress response phenotype. In the literature, it has already been suggested that primiparous cows could be less sensitive to heat stress than multiparous cows^27,29,30^ which is consistent with our results. Concerning the period of lactation, the fixed effect solutions obtained increase quickly at the beginning of lactation to reach the maximum around 20–50 DIM. Then, it started to slowly decrease to reach the minimum at the end of lactation. This corresponds to the higher susceptibility to heat stress of cows in early lactation compared to cows in late lactation reported in the literature^5,30,31^. Finally, it is well accepted that cows with the highest yields are the most affected by heat stress which was also highlighted by the solutions per milk yield class in this study.

Globally, even if no formal validation was performed, those results support the idea that the FT-MIR prediction for the combined heat stress response behaves in a consistent, relevant, expected and explainable manner as wanted for a good heat stress response prediction. Several other studies already highlighted the impact of heat stress on various milk components including MIR-predicted markers^10,18,26,32^, which supports our findings that MIR spectra can reflect the heat stress status of dairy cows. However, to our knowledge, none has directly used FT-MIR spectra to predict this status. Yet, using FT-MIR spectra is of great interest because it can be implemented immediately in routine without any additional measurements or costs, unlike methods requiring direct physiological information. A future perspective of this work is to improve the prediction equation with new data from a wider range of farms and to adapt it for future in-line applications enabling a real-time monitoring of the cows’ heat stress status^33^.

## Conclusion

A first prediction equation to record individual heat stress responses of dairy cows based on milk FT-MIR spectra was developed in this study. This approach enables heat stress response recording in routine without additional measurements and costs. The model selected was the combination of two models based on two definitions of the response to heat stress and the prediction presented coherent responses to different parameters. Indeed, the large-scale assessment on external data presented expected patterns with the THI classes as well as with cow characteristics. This study also showed the diversity of responses between cows in the same environmental conditions as well as between farms with similar THI measurements.

## Data Availability

The datasets generated during the current study are available from the corresponding author on reasonable request. Historical milk recording data from the Walloon Breeders Association (awé groupe – Elevéo) (Ciney, Belgium) are not publicly available due to the private nature of these data. They are however available from the corresponding author upon reasonable request and permission of the Walloon Breeders Association.
