# Correction: Prediction of heat stress response in dairy cows using milk mid-infrared spectra

**DOI:** 10.1038/s41598-026-60149-x

**Published:** 2026-07-02

**Authors:** Pauline Lemal, Clément Grelet, Frédéric Dehareng, Hélène Soyeurt, Martine Schroyen, Nicolas Gengler

**Affiliations:** 1 University of Liège, Gembloux Agro-Bio Tech (ULiège-GxABT), Gembloux, 5030 Belgium; 2 Walloon Agricultural Research Center (CRA-W), Gembloux, 5030 Belgium

Correction to: *Scientific Reports* 10.1038/s41598-026-39287-9, published online 19 March 2026

The original version of this Article contained an error in Table 1, where the numbers in the last line were inadvertently shifted one column to the left.

The original Table 1 and its accompanying legend appear below.

**Table 1:**
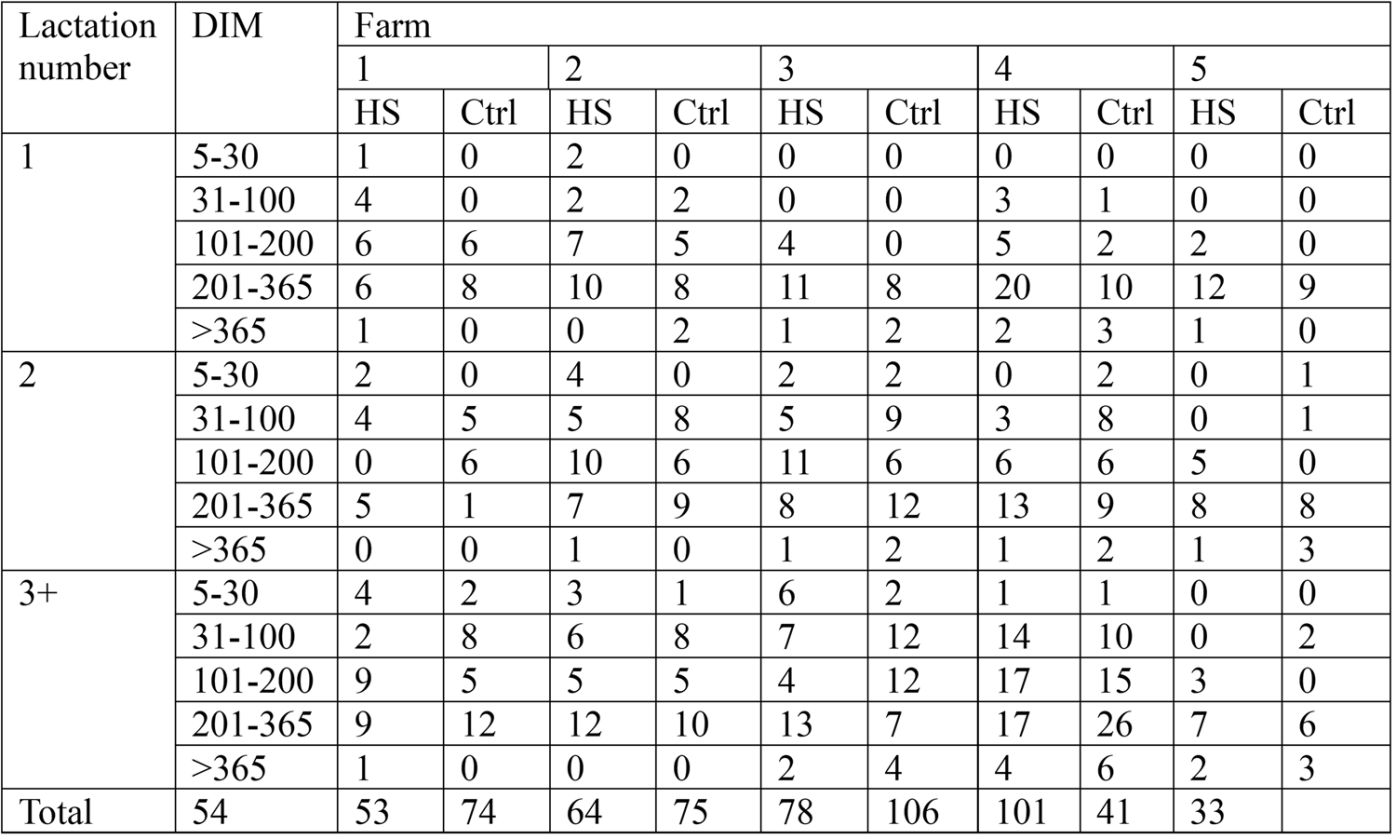
Distribution of sampled animals for surface body temperature simultaneously with a milk recording.

Data collected during a heat wave are shown in the HS columns and data recorded during a thermoneutral period are presented in the Ctrl columns.

The original Article has been corrected.

